# Intentional Modulation of Ibrutinib Pharmacokinetics through CYP3A Inhibition

**DOI:** 10.1158/2767-9764.CRC-21-0076

**Published:** 2021-11-09

**Authors:** Eric D. Eisenmann, Qiang Fu, Elizabeth M. Muhowski, Yan Jin, Muhammad Erfan Uddin, Dominique A. Garrison, Robert H. Weber, Jennifer A. Woyach, John C. Byrd, Alex Sparreboom, Sharyn D. Baker

**Affiliations:** 1 Division of Pharmaceutics and Pharmacology, College of Pharmacy, The Ohio State University, Columbus, Ohio.; 2 Division of Hematology, Department of Internal Medicine, College of Medicine, The Ohio State University, Columbus, Ohio.

## Abstract

Ibrutinib (Imbruvica; PCI-32765) is an orally administered inhibitor of Bruton's tyrosine kinase that has transformed the treatment of B-cell malignancies. However, ibrutinib has very low oral bioavailability that contributes to significant variability in systemic exposure between patients, and this has the potential to affect both efficacy and toxicity. We hypothesized that the oral bioavailability of ibrutinib is limited by CYP3A isoform–mediated metabolism, and that this pathway can be inhibited to improve the pharmacokinetic properties of ibrutinib. Pharmacokinetic studies were performed in wild-type mice and mice genetically engineered to lack all CYP3A isoforms (CYP3A^−/−^) that received ibrutinib alone or in combination with CYP3A inhibitors cobicistat or ketoconazole. Computational modeling was performed to derive doses of ibrutinib that, when given after a CYP3A inhibitor, results in therapeutically relevant drug levels. Deficiency of CYP3A in mice was associated with an approximately 10-fold increase in the AUC of ibrutinib. This result could be phenocopied by administration of cobicistat before ibrutinib in wild-type mice, but cobicistat did not influence levels of ibrutinib in CYP3A^−/−^ mice. Population pharmacokinetic and prospectively validated physiologically based pharmacokinetic models established preclinical and clinical doses of ibrutinib that could be given safely in combination with cobicistat without negatively affecting antileukemic properties. These findings signify a dominant role for CYP3A-mediated metabolism in the elimination of ibrutinib, and suggest a role for pharmacologic inhibitors of this pathway to intentionally modulate the plasma levels and improve the therapeutic use of this clinically important agent.

**Significance:**

Ibrutinib has limited oral bioavailability, which contributes to significant interindividual pharmacokinetic variability. Using engineered mouse models, we here report a causal relationship between CYP3A-mediated metabolism and ibrutinib's bioavailability and drug–drug interaction with cobicistat. These results offer a mechanistic basis for reported pharmacokinetic interactions with ibrutinib, and in conjunction with a newly developed computational model, allow for the rational design of clinical trials aimed at improving the therapeutic use of ibrutinib.

## Introduction

Ibrutinib is an orally administered tyrosine kinase inhibitor (TKI) targeting Bruton's tyrosine kinase (BTK) that has transformed the treatment for cancers driven by B-cell proliferation (1). Despite improving patient outcomes, ibrutinib has a highly variable pharmacokinetic profile that could predispose patients to excess or insufficient drug levels, which in turn can lead to adverse effects or treatment failure, respectively (2). The need to further understand the mechanisms underlying this unpredictable pharmacokinetic profile is underscored by the notion that more than 10% of patients are eventually taken off ibrutinib therapy due to unacceptable adverse events (3).

Unique among FDA-approved TKIs, the oral bioavailability of ibrutinib is very low (<3%), and this unfavorable feature is an important contributor to the significant interindividual variability in drug exposure observed in patients (4, 5). Given that ibrutinib is nearly completely absorbed after oral administration, the low bioavailability of ibrutinib is likely due to extensive first-pass metabolism mediated by isoforms of the drug-metabolizing enzyme cluster CYP3A (6). Support for this hypothesis comes from clinical pharmacokinetic studies indicating that coadministration of ibrutinib with ketoconazole, an antifungal drug that potently inhibits CYP3A isoforms, results in a 24-fold increase in the area under the concentration–time curve (AUC) of ibrutinib and a decreased exposure to the main CYP3A-mediated metabolite, PCI-45227 (7). Increases in the exposure to ibrutinib have also been observed following coadministration of other CYP3A inhibitors, including itraconazole (8), erythromycin and voriconazole (9), and the implications for ibrutinib dose modification have been documented (10, 11). Considering the sensitivity of ibrutinib to CYP3A-mediated metabolism *in vitro* (12) and *in vivo* (13), unintentional CYP3A inhibition could potentially cause dangerous increases in ibrutinib exposure when ibrutinib is administered at standard doses. However, intentional inhibition of CYP3A activity could theoretically be harnessed to deliberately increase ibrutinib bioavailability and allow for the administration of substantially lower ibrutinib doses that achieve effective drug levels with greater consistency and reduced pharmacokinetic variability (14). This so-called boosting concept is already commonly applied in several therapeutic indications, including for the treatment of HIV and Parkinson's disease, but is rarely used in cancer (15).

The aim of this study was to develop a pharmacokinetic boosting strategy for ibrutinib. To achieve this, we used genetically engineered knockout mice to unambiguously define the contribution of CYP3A to the oral bioavailability of ibrutinib and examined the specificity of various boosting agents to intentionally modulate the pharmacokinetic profile of ibrutinib. Finally, we used computational modeling approaches to determine the feasibility of boosting a low dose of ibrutinib to therapeutically relevant levels in plasma with extrapolation to a clinical scenario.

## Materials and Methods

### Chemical and Reagents

Ibrutinib was purchased from LC Labs. Cobicistat and ketoconazole were purchased from MedChemExpress. Reference standards of ibrutinib, its primary metabolite PCI-45227 (M37) and their corresponding deuterated versions, used as internal standards for the analytic method, were purchased from AlsaChim. PEG300, PEG400, corn oil, and polysorbate-80 (Tween-80) were purchased from Sigma-Aldrich, and LC-MS–grade formic acid (FA), methanol, and acetonitrile were from Thermo Fisher Scientific. Blank plasma was obtained from FVB wild-type mice (Taconic Biosciences). The following antibodies were obtained from Cell Signaling Technology: anti-BTK (catalog no. 8547). Anti-phospho-BTK (Y223, catalog no. ab68217) was purchased from Abcam and anti-GAPDH (catalog no. MAB374) was purchased from Millipore Sigma.

### *In Vivo* Pharmacokinetic Studies

Experiments were performed with male and female mice from inbred wild-type strains (FVB, C57BL/6, DBA) or with age- and sex-matched genetically engineered mice with a deficiency of the *Cyp3a* locus [(8 genes; FVB.129P2-*Cyp3a13^tm1Ahs^* Del (5Cyp3a57-Cyp3a59)1Ahs); ref. 16; CYP3A^−/−^], a deficiency of the intestinal transporter OATP2B1 (ref. 17; OATP2B1 ^−/−^), or the hepatic transporter OATP1B2 (ref. 18; OATP1B2 ^−/−^). All mice used had a body weight of 20–30 g at an age of 10–18 weeks. The studies were approved by the University Laboratory Animal Resources Animal Care and Use Committee at The Ohio State University (Columbus, OH). Mice were housed in a temperature- and light-controlled environment with free access to water and a standard diet, excluding a 3-hour fast before ibrutinib administration. For *in vivo* studies, drugs were prepared in standard vehicles (19, 20). Details regarding the preparation of drugs are provided in the Supplementary Methods.

Pharmacokinetic studies were performed as described previously (21). Further details are provided in the Supplementary Methods.

### Microsomal Experiments

Microsomes were prepared from liver and duodenum (*n* = 3 per group) obtained from both male and female FVB wild-type and CYP3A^−/−^ littermates using standard procedures. To determine the rate of PCI-45227 formation from ibrutinib in the liver and intestinal microsomal preparations, ibrutinib (10 μmol/L) was incubated with 100 μg of microsomal suspensions for 1 hour. Details regarding the isolation of microsomes are provided in the Supplementary Methods.

### Quantitative Measurement of Ibrutinib and PCI-45227 Concentrations

Ibrutinib and PCI-45227 were quantified using a validated ultra–high-performance LC/MS-MS analytic method. The lower limit of quantification was 5 ng/mL, using 5-μL sample volumes. Details regarding the analytic method are provided in the Supplementary Methods.

### Pharmacokinetic Data Analysis

Noncompartmental analysis using Phoenix WinNonlin version 8.0 (Certara) was used to derive pharmacokinetic parameters. The maximum plasma concentration (*C*_max_) was determined by visual inspection of the data from the concentration–time curves. The linear trapezoidal rule was used to obtain the AUC over the sample collection interval. Oral bioavailability (F) was calculated by dividing the AUC obtained with oral administration (AUC_oral_) by the AUC obtained with intravenous administration of ibrutinib alone (AUC_iv_) after correction for dose differences. The relative heart exposure was expressed as the heart-to-plasma ratio, calculated by determining the ibrutinib concentration in the heart (ng/mg tissue), corrected for contaminating blood, and dividing by the corresponding ibrutinib concentration in plasma (ng/mL plasma) at the same collection timepoint.

### Computational Modeling

Nonlinear mixed-effect model development was performed using NONMEM software version 7.3 and PDx-Pop 5.0 (ICON Development Solutions). Postprocessing of graphical plots in NONMEM was performed using Rstudio version 1.14 (Rstudio). The population pharmacokinetics of ibrutinib and its metabolite PCI-45227 were analyzed in a stepwise manner. All models were created using the first-order conditional estimation method, and the subroutine ADVAN5 was used to describe all model kinetics processes, which included interindividual and residual variabilities. The model determination was guided by the minimum objective function value, the Akaike Information Criterion, and visual inspection of diagnostic plots. The best structural model for ibrutinib involved first-order absorption in a two-compartment pharmacokinetic model, while PCI-45227 data were fitted to a one-compartment model with first-order disposition. A physiologically based pharmacokinetic (PBPK) model for ibrutinib and cobicistat was constructed using a Simcyp population-based ADME simulator (V19; Simcyp Limited). The drug-dependent properties included the parameters necessary to describe the ADME processes and the drug–drug interaction mechanisms. The pharmacokinetics of ibrutinib after administration of a single oral dose were described by a minimal PBPK model and the ADAM model (22). Model parameters were taken from the published literature (23, 24) as well as from Drugbank (Supplementary Table S1).

### *In Vitro* Transport Assays

Transport assays assessing uptake of ibrutinib by cells overexpressing drug transporters were conducted as described previously (25). In general, cells overexpressing transporters of interest (OATP1B1, OATP1B3, OATP2B1, OCT2, MATE1, OCT1, or OCT3) or the corresponding empty vector control (VC) were seeded and incubated at 37°C for 24 hours. Cells were then incubated with the indicated substrate for 15 minutes. The experiment was then terminated and the cells were lysed. Total protein was measured using a microplate spectrophotometer. Intracellular drug concentrations were estimated in the remaining cell lysate by liquid scintillation counting. Transporter-mediated uptake was calculated by dividing the disintegrations per minute (dpm) from each replicate by the amount of protein (mg), and subtracting the dpm/mg protein in the respective VC cell line from the dpm/mg protein in the transporter-overexpressed cells.

### Cell Culture and Viability Assays

Blood was drawn from patients with newly diagnosed, untreated CLL after receipt of written informed consent under a protocol approved by the Institutional Review Board of The Ohio State University (Columbus, OH) in accordance with the Declaration of Helsinki. B cells were isolated from whole blood using Rosette-Sep B (StemCell Technologies). Primary CLL cells were cultured in RPMI1640 medium supplemented with 10% FBS, 100 U/mL penicillin, 100 μg/mL streptomycin, and 2 mmol/L l-glutamate. Cells were treated with DMSO, ibrutinib (1 μmol/L), cobicistat (1, 10 μmol/L) or ibrutinib (1 μmol/L) with cobicistat (1, 10 μmol/L). After 1 hour, the cells were pelleted and resuspended in fresh media. For experiments with extended drug exposure, DMSO and cobicistat were added again after washout.

The human lymphoma TMD8 (RRID:CVCL_A442) cell line (kindly provided by Dr. Sriram Balasubramanian from Janssen Pharmaceuticals in 2015, authenticated by Ion Torrent Panel, and verified to be *Mycoplasma* free) was maintained in RPMI (Thermo Fisher Scientific) with 10% FBS. Cells were used within 30 passages after thawing and were routinely checked to ensure there was no *Mycoplasma* contamination (MycoAlert Detection Kit). Drug treatment effects on these cells were assessed by an MTT assay (Roche Diagnostics). Cells were seeded in a 96-well plate at a final concentration of 150,000 cells/mL and incubated with ascending concentrations of ibrutinib with or without cobicistat or ketoconazole. Cell viability was assessed after 72 hours of incubation. Surface–response analysis was performed using the Combenefit software (26).

### Immunoblotting

Primary CLL cells treated with ibrutinib and/or cobicistat were stimulated by spinning onto a 6-well plate coated with anti-IgM antibody (Jackson ImmunoResearch Laboratories) at a concentration of 10 μg/mL. After 15 minutes of stimulation, cell lysates were collected and analyzed by SDS-PAGE. Protein was transferred to nitrocellulose and blots were probed with primary antibodies and horseradish peroxidase (HRP)-conjugated secondary antibodies. Blots were imaged using WesternBright chemiluminescent substrate (Advansta) on an X-ray film. Phosphorylated proteins were analyzed first, then antibodies were removed using Western Blot Stripping Buffer (Thermo Fisher Scientific) before analyzing total protein. Membranes were stripped after imaging total proteins before analyzing loading control. DC9 cell line lysates were used as positive controls. Loading controls were assessed for each membrane and are presented with corresponding proteins analyzed on the same membrane.

### Statistical Analysis

All data represent mean and SEM, unless stated otherwise. For pharmacokinetic studies comparing only two groups (i.e., ketoconazole vs. vehicle, boosted 4 mg/kg ibrutinib vs. unboosted 30 mg/kg ibrutinib), unpaired *t* tests were performed comparing the *C*_max_ and AUC of each treatment group. For mechanistic studies with cobicistat, a two-way ANOVA adjusted using Tukey multiple comparison test was performed with genotype (wild-type or CYP3A^−/−^) and treatment group (none, vehicle, or CYP3A inhibitor) as between-subjects variables to determine main effects and to compare the *C*_max_ and AUC of each treatment group when ibrutinib was dosed orally. A similar two-way ANOVA was performed with intravenous ibrutinib data. An unpaired *t* test was used to compare the mean heart-to-plasma ratio between vehicle- and cobicistat-treated mice and the pharmacokinetic parameters derived from the PBPK model. Statistical analysis was performed using GraphPad Prism 9.0 (GraphPad Software Inc.). All statistical tests were two-tailed, and *P* < 0.05 was considered statistically significant.

### Data Availability

The data generated in this study are available upon request from the corresponding author.

## Results

### Influence of Pharmacologic CYP3A Inhibitors on Ibrutinib Exposure

**FIGURE 1:**
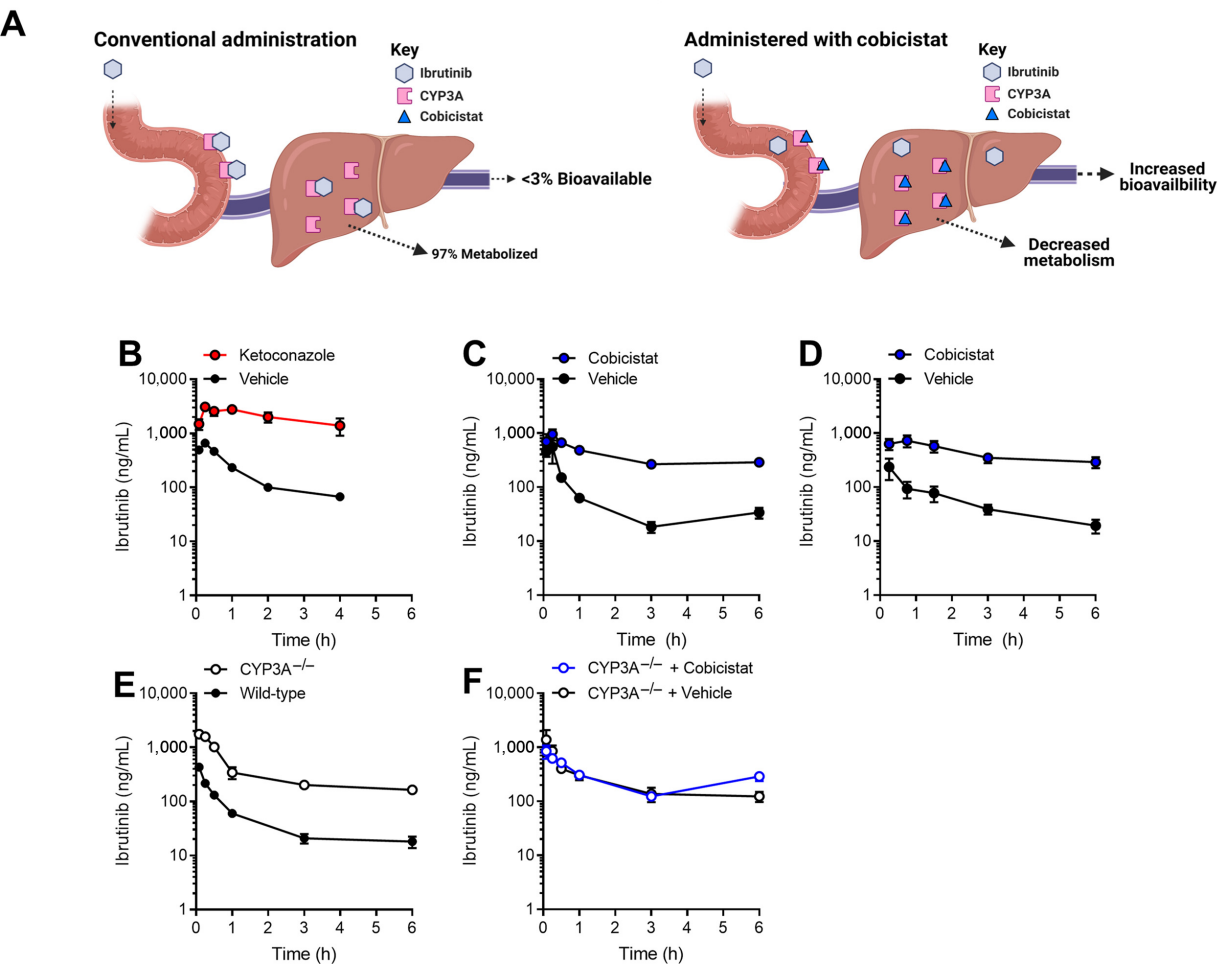
Pharmacologic and genetic inhibition of CYP3A increases ibrutinib exposure. **A,** Proposed mechanism of interaction to boost ibrutinib exposure with inhibitors of CYP3A-mediated metabolism such as cobicistat. When administered alone (left), the oral bioavailability of ibrutinib is limited by extensive metabolism. When CYP3A is inhibited (right), ibrutinib bioavailability is increased. Diagram created with BioRender.com. **B–D,** Plasma concentrations of ibrutinib in wild-type mice receiving oral ibrutinib (10 mg/kg) 30 minutes after oral ketoconazole (50 mg/kg; **B**) or cobicistat (30 mg/kg) followed by a single dose of ibrutinib (**C**) or after 5 consecutive daily doses (**D**). **E** and **F,** Plasma concentrations of ibrutinib in wild-type and CYP3A^−/−^ mice (**E**) and in CYP3A^−/−^ mice receiving oral ibrutinib (10 mg/kg) 30 minutes after vehicle or cobicistat (30 mg/kg; **F**). Data presented represent the mean (symbols) ± SEM (error bars) of 4–10 observations per group.

Given the known safety concerns associated with adding boosting agents, such as the CYP3A inhibitor ketoconazole, to the standard doses of ibrutinib in human patients (8), we first sought to determine whether mice could be used as a translationally relevant model to study the influence of CYP3A inhibitors and genotype status on the pharmacokinetics of ibrutinib (Fig. 1A). We treated female wild-type mice with oral ibrutinib (10 mg/kg) 30 minutes after a single oral dose of ketoconazole (50 mg/kg; ref. 27) or vehicle. Ketoconazole pretreatment in female mice was associated with significant increases in exposure to ibrutinib by about 11-fold compared with controls (*P* < 0.01; Fig. 1B). In support of the thesis that this ketoconazole–ibrutinib interaction is mechanistically linked with inhibition of CYP3A, we found that levels of PCI-45227, the main CYP3A-mediated metabolite of ibrutinib, and the metabolic ratio of PCI-45227 to the parent drug were substantially decreased by ketoconazole pretreatment (Supplementary Fig. S1A). This degree of interaction is similar to that reported previously for ketoconazole in a clinical study (7), suggesting that mice could serve as an appropriately predictive model for ibrutinib pharmacokinetics in humans.

In view of the previously reported safety concerns associated with the use of ketoconazole (28) and its relatively poor selectivity for CYP3A-mediated mechanisms (29), we next performed similar experiments with cobicistat, a more selective CYP3A inhibitor that is approved as a boosting agent for certain drugs used in the treatment of HIV (30, 31). In addition, compared with azole antifungals, cobicistat is well tolerated (30), does not cause liver dysfunction (32) or cardiac dysfunction (33), and can therefore be considered a preferred boosting agent. Under conditions similar to those used for ketoconazole, we found that cobicistat increased the exposure to ibrutinib by about 9-fold (*P* < 0.01), regardless of whether ibrutinib was given as a single dose (Fig. 1C) or at steady-state with daily oral dosing of ibrutinib and analysis performed on day 5 (Fig. 1D). Concurrent with these changes in ibrutinib levels, cobicistat significantly decreased the levels of PCI-45227 and the PCI-45227-to-ibrutinib ratio, indicating that cobicistat suppressed the function of CYP3A (Supplementary Fig. S1B and S1C).

### Dependence of Ibrutinib Oral Bioavailability on CYP3A Function

To unambiguously determine the contribution of CYP3A to the pharmacokinetic profile of ibrutinib, we next performed studies in mice lacking the 8 murine *Cyp3a* genes (CYP3A^−/−^; ref. 16). Compared with wild-type mice, the ibrutinib AUC was increased about 8-fold in CYP3A^−/−^ mice with almost undetectable levels of PCI-45227 (*P* < 0.01; Fig. 1E). To unequivocally demonstrate that the influence of cobicistat on ibrutinib exposure is exclusively dependent on an effect on CYP3A activity, we next treated CYP3A^−/−^ mice with ibrutinib in the presence or absence of cobicistat pretreatment. These studies indicated that the pharmacokinetic profile of ibrutinib was similar in CYP3A^−/−^ mice regardless of cobicistat coadministration (*P* > 0.05; Fig. 1F). To discriminate between absorption and disposition as the cobicistat-sensitive process in the pharmacokinetics of ibrutinib, we also administered ibrutinib intravenously to wild-type and CYP3A^−/−^ mice that had been pretreated with oral cobicistat or vehicle. In this experiment, ibrutinib exposure was only increased 2-fold by CYP3A deficiency, and similarly, cobicistat increased the exposure to ibrutinib in wild-type mice by 2-fold. However, cobicistat did not further increase exposure to ibrutinib in the absence of CYP3A (Supplementary Fig. S2). Together, these results suggest that cobicistat-induced increases in ibrutinib exposure are predominantly mediated by effects on CYP3A-mediated first-pass metabolism (Table 1). Comparative *ex vivo* incubations of ibrutinib with intestinal and hepatic microsomal preparations from wild-type mice and CYP3A^−/−^ mice (Supplementary Fig. S3) indicated a preferential formation of PCI-45227 by CYP3A-mediated metabolism in the liver, suggesting this is the likely site of interaction with cobicistat. This observation is in line with results from a clinical study indicating that the plasma concentrations of ibrutinib are not substantially altered by intake of grapefruit juice (34), which can cause drug–food interactions through inhibition of intestinal but not hepatic CYP3A4 function (35).

**TABLE 1:** Ibrutinib and PCI-45227 pharmacokinetic parameters.

Mouse genotype	Ibrutinib dose (mg/kg)	Cotreatment (dose mg/kg)	Ibrutinib *C*_max_ (ng/mL)	Ibrutinib AUC(0-last) (ng/h/mL)	F (%)	Ibrutinib AUC fold increase	PCI-45227 *C*_max_ (ng/mL)	PCI-45227 AUC(0-last) (ng/h/mL)	PCI-45227: Ibrutinib AUC ratio
Wild-type FVB	10	PEG400	663 (328)	768 (147)	NC	—	246 (54)	668 (155)	0.87
Wild-type FVB	10	Ketoconazole (50)	3,105 (404)^a^	8,315 (1,543)^a^	NC	10.8	288 (89)	682 (250)	0.08
Wild-type FVB	10	Corn oil	292 (79)	284 (41)	5.5	—	219 (61)^b^	563 (91)^d^	2.0
Wild-type FVB	10	Cobicistat (30)	1,438 (567)	2,599 (471)^c^	50	9.2	45 (14)^c^	139 (56)^c^	0.05
Wild-type FVB	10	Corn oil^e^	394 (72)	459 (59)	8.8	—	405 (74)	917 (128)	2.0
Wild-type FVB	10	Cobicistat^e^ (30)	925 (220)	2,254 (335)^c^	43	4.9	211 (65)	560 (73)^b^	0.25
Wild-type FVB	10	None	430 (63)	301 (38)	5.8	—	447 (45)	765 (79)	2.5
CYP3A^−/−^	10	None	1,762 (229)	2,347 (203)^c^	45	7.8	8.2 (2.1)^c^	6.3 (1.7)^c^	0.003
CYP3A^−/−^	10	Corn oil	1,389 (698)	1,410 (260)	27	—	ND	ND	ND
CYP3A^−/−^	10	Cobicistat (30)	845 (230)	1,660 (131)^d^	32	1.2	ND	ND	ND
Wild-type FVB	30	None	554 (107)	1,713 (163)	11	—	837 (77)	3,281 (154)	1.9
Wild-type FVB	4	Cobicistat (30)	589 (105)	1,202 (142)^b^	58	0.7	30.7 (12.1)^c^	56 (22)^c^	0.05

NOTE: Values are the mean with SE in parenthesis. Treatment groups involved 5–10 female mice per experiment.

Abbreviations: NC, not calculated (different terminal timepoint than IV study); ND, not determined (below the analytic assay lower limit of quantitation).

^a^P < 0.01.

^b^P < 0.05.

^c^P < 0.001.

^d^Not significant.

^e^Steady-state.

Given that the pharmacokinetic profile of ibrutinib was previous found to be sexually dimorphic in rats (36), we performed similar experiments with ketoconazole or cobicistat in male FVB wild-type mice. Administration of ketoconazole or cobicistat resulted in 10-fold and 7-fold increases in ibrutinib AUC in male mice, respectively (Supplementary Fig. S4), in line with observations made in female mice. Given that the metabolite-to-parent ratios were similar in both male and female mice, subsequent studies were only performed in female mice. Notably, regardless of sex, the pharmaceutical excipient present in the formulation of CYP3A inhibitors did not significantly alter levels of ibrutinib, suggesting that the observed pharmacokinetic alterations were entirely mediated by the perpetrating drugs themselves (Supplementary Fig. S4; Supplementary Table S2).

### Effects of Xenobiotic Transporters on Ibrutinib Pharmacokinetics

To verify the specificity of cobicistat for CYP3A-mediated metabolism of ibrutinib, several additional studies were performed to rule out contributions of important xenobiotic uptake transporters in organs of absorption or elimination to the observed findings. Incentive for these studies was provided by the notion that the pharmacokinetic profile of several TKIs is rate-limited by such transporters (16, 33) and by prior data indicating that many potent CYP3A inhibitors, including cobicistat, have a propensity to also inhibit drug transporters (14). Initial studies were performed in heterologous expression models engineered to overexpress the hepatic uptake transporters OATP1B1 and OATP1B3, the renal transporters OCT2 and MATE1, or the intestinal transporters OATP2B1 and OCT3. Compared with positive control substrates for which transporter overexpression resulted in a more than 5-fold increase in uptake (Supplementary Fig. S5A), the uptake of ibrutinib was not substantially facilitated by any of the tested transporters (Supplementary Fig. S5B). Because the exclusive dependence on *in vitro* models can result in false-negative observations, we next examined a potential *in vivo* contribution of select transporters to the pharmacokinetic of ibrutinib. These studies were performed in mice with a genetic deficiency of OATP1B2, the murine ortholog of human OATP1B1 and OATP1B3, or OATP2B1, because these transporters were previously found to be relevant to the absorption or disposition of related TKIs (16, 33). Consistent with CYP3A-mediated metabolism being the primary contributor to the low oral bioavailability of ibrutinib, we found that the systemic levels of ibrutinib were not altered in mice lacking OATP1B2 or OATP2B1 (Supplementary Fig. S5C–S5E; Supplementary Table S2), although levels of PCI-45227 were decreased in mice lacking OATP2B1 (*P* < 0.05). These findings are consistent with data included in the prescribing information for ibrutinib (6) indicating that the agent is not a transported substrate of these uptake carriers.

### Influence of Cobicistat on Cardiac Distribution of Ibrutinib

Given the established risk of cardiotoxicity associated with ibrutinib (37) and the ability of uptake transporters to impact the distribution of xenobiotics into cardiomyocytes (38), we next sought to determine whether cobicistat altered exposure of the heart to ibrutinib. Interestingly, the hearts of mice pretreated with cobicistat had substantially lower levels of ibrutinib than mice pretreated with vehicle, when normalized to the levels of ibrutinib present in plasma (*P* < 0.01; Supplementary Fig. S6). This finding is consistent with the ability of cobicistat to potently inhibit the function of several uptake transporters (39) that are expressed in the mammalian heart (38), and lends support to the thesis that the cardiac accumulation of ibrutinib is mediated by a presently unidentified, cobicistat-sensitive transporter.

### Effects of Cobicistat on the Antileukemic Properties of Ibrutinib

**FIGURE 2:**
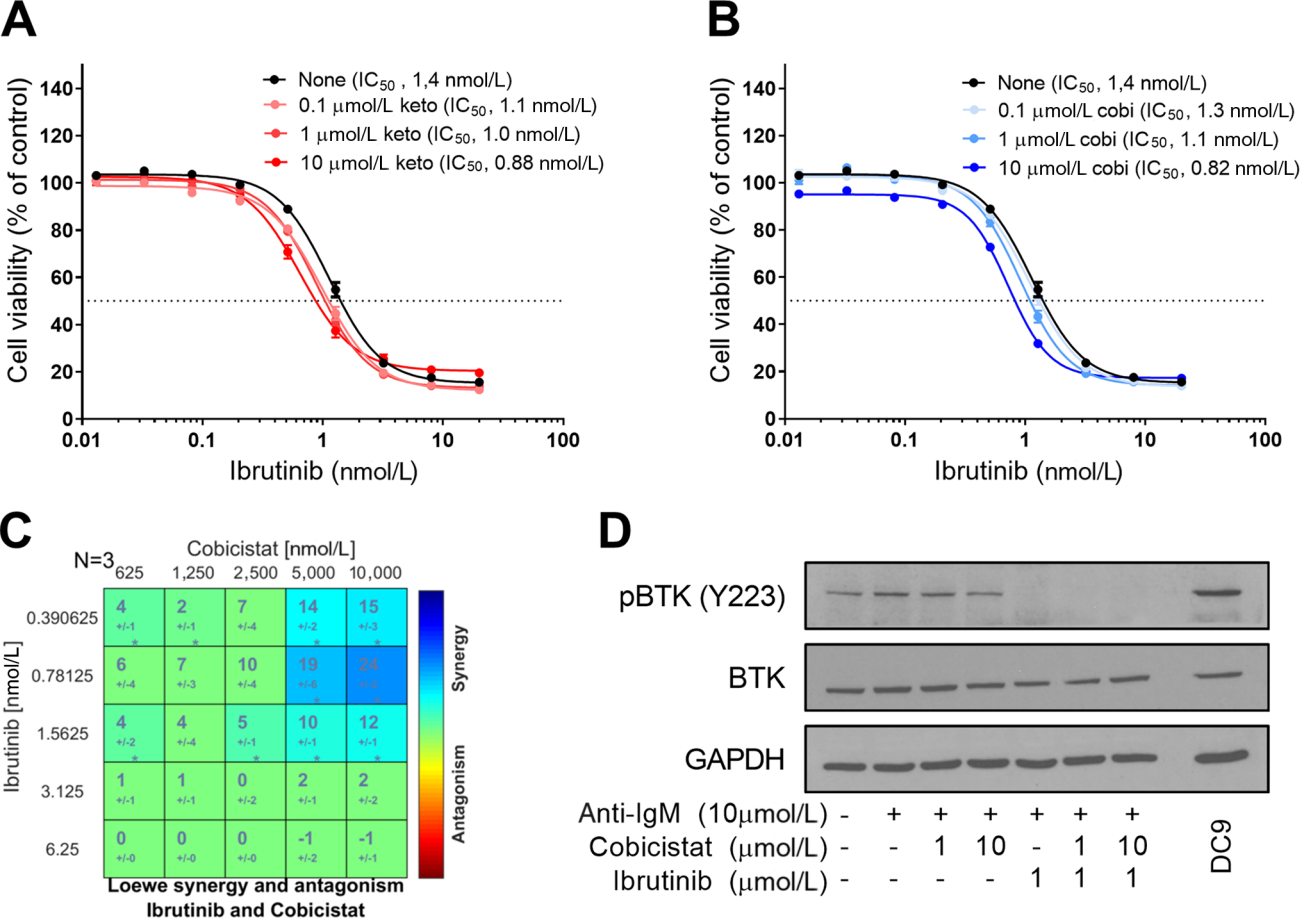
CYP3A inhibitors do not influence the antileukemic activity of ibrutinib. TMD8 cells were treated with increasing ibrutinib concentrations in the absence and presence of ketoconazole (**A**) or cobicistat (**B**) for 72 hours followed by MTT assay (*n* = 18–24 in 3–4 biological replicates). **C,** Synergy distribution when TMD8 cells were treated with ibrutinib and cobicistat at fixed ratios and analyzed using Combenefit (*n* = 9 in three biological replicates). Viability data presented represent the mean (symbols) ± SEM (error bars) of 18–24 observations in 3–4 biological replicates per group. **D,** Primary CLL cells were treated with ibrutinib and/or cobicistat followed by immunoblotting. Experiment repeated four times each with a unique patient sample. Blot shown is from one patient and representative of all four.

Although combining ibrutinib with cobicistat could possibly be employed as a boosting strategy to improve the pharmacokinetic properties of ibrutinib and reduce exposure to the heart as an important site of injury, it is important to establish that the antileukemic properties of ibrutinib are not compromised by the concurrent administration of CYP3A inhibitors. Indeed, the success of an effective combination boosting therapy would depend on the selected inhibitor, on dosing/scheduling strategies, on intrinsic anticancer properties of the boosting agent, and on the expression status of uptake and efflux transporters in malignant cells that are sensitive to modulation by the boosting agent. To gain preliminary insight, we performed *in vitro* viability assays with ibrutinib-responsive TMD8 B-cell lymphoma cells treated for 72 hours with ibrutinib and a fixed concentration (0.1 μmol/L, 1 μmol/L, or 10 μmol/L) of ketoconazole (Fig. 2A) or cobicistat (Fig. 2B). These assays demonstrated that neither ketoconazole nor cobicistat negatively influenced the antileukemic activity of ibrutinib. We then performed combinatorial MTT assays with varying ratios of ibrutinib and cobicistat analyzed utilizing response surface techniques (26). This analysis demonstrated a positive synergy distribution (Fig. 2C) and suggests that the addition of pharmacologic inhibitors of CYP3A could potentially enhance the antileukemic efficacy of ibrutinib. On the basis of these data, we collected B cells from patients with previously untreated CLL and treated these primary samples with ibrutinib and/or cobicistat. Immunoblotting demonstrated that the addition of cobicistat did not impact ibrutinib's ability to suppress BTK signaling (Fig. 2D).

### Computational Model-Based Predictions of Ibrutinib Pharmacokinetics

**FIGURE 3:**
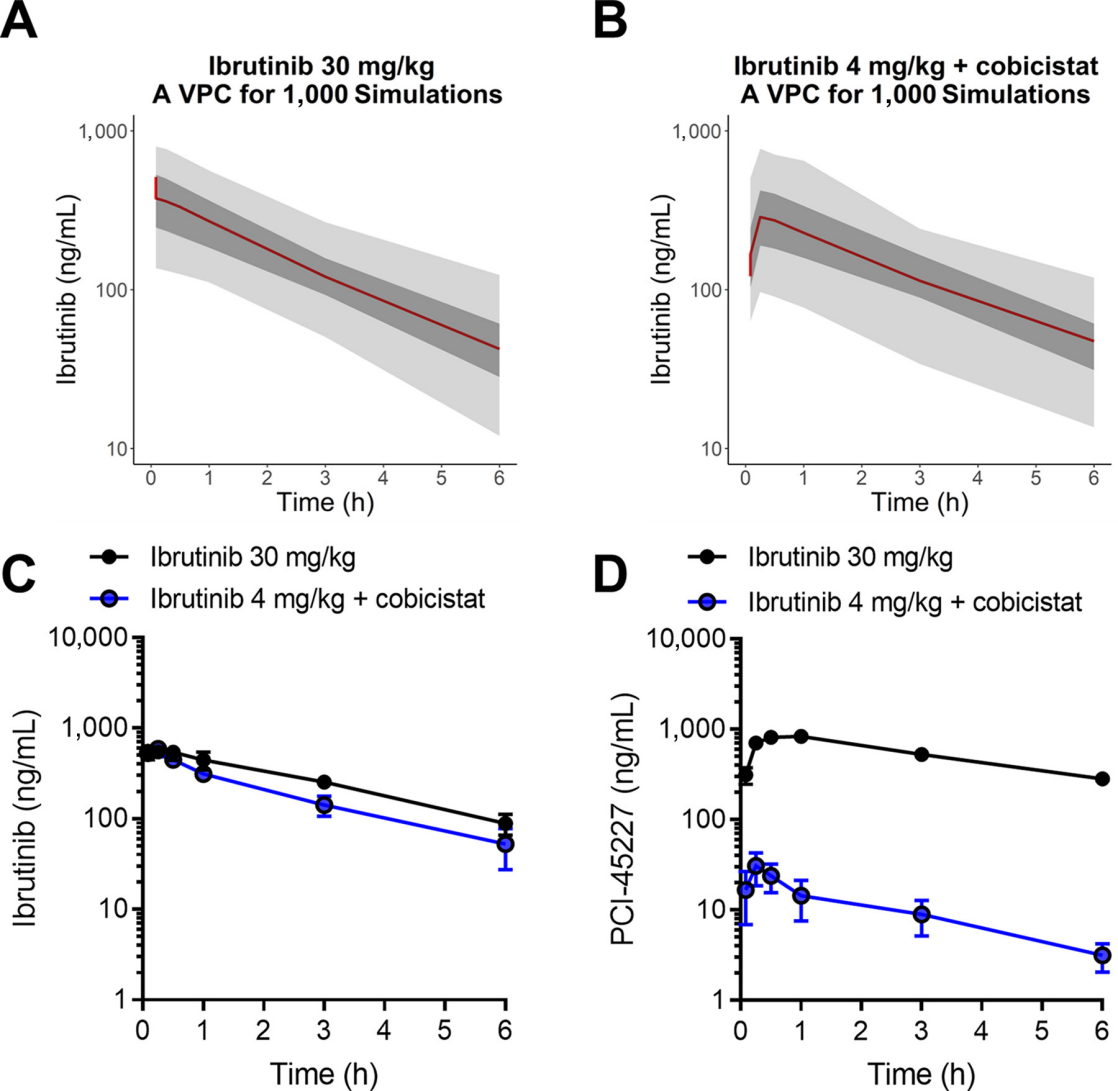
Pharmacokinetic modeling to predict ibrutinib exposure with and without cobicistat administration. **A** and **B,** NONMEM-based predicted concentration–time curves of ibrutinib from 1,000 simulations following administration of ibrutinib 30 mg/kg alone (**A**) or ibrutinib 4 mg/kg with cobicistat 30 mg/kg (**B**). The red lines are median concentrations, the 25th–75th percentile is shown in dark gray and the 5th–95th percentile in light gray. **C** and **D,** Experimentally derived plasma concentration–time curves of ibrutinib (**C**) and PCI-45227 (**D**) in wild-type mice receiving the model-predicted ibrutinib doses of 30 mg/kg alone or 4 mg/kg 30 minutes after cobicistat (30 mg/kg). Data presented represent the mean (symbols) ± SEM (error bars) of five observations per group.

Mechanistic modeling and simulation approaches are frequently utilized to predict drug–drug interactions and describe relationships between drug exposure and pharmacodynamic endpoints (40, 41). This type of strategy has previously been utilized with clinical data to predict changes in exposure to ibrutinib in the context of concurrent administration of CYP3A inhibitors (23). On the basis of our hypothesis and initial experiments (Fig. 3A), we next developed a nonlinear mixed-effect population pharmacokinetic model and performed simulations to determine a cobicistat-boosted ibrutinib dose that is equivalent to an unboosted ibrutinib dose of 30 mg/kg, which was previously found to be effective in murine models of disease (2, 6). This model predicted that an ibrutinib dose of 4 mg/kg preceded by cobicistat would achieve measures of systemic exposure similar to those associated with ibrutinib given alone at a dose of 30 mg/kg (Fig. 3B; Supplementary Table S3). Gallais and colleagues previously reported that first-pass metabolism of ibrutinib in humans can be modeled by a link between the dosing compartment and metabolite central compartment (42). However, our model was unable to link ibrutinib and metabolite levels due to the lack of detectable metabolite levels when CYP3A was genetically or pharmacologically inhibited. The validity of this population pharmacokinetic model was determined by goodness-of-fit analyses, and formed the basis of a prospective validation study comparing the pharmacokinetic profile of ibrutinib with the drug given alone at 30 mg/kg or at 4 mg/kg after pretreatment with cobicistat (Fig. 3C and D). The observed findings were consistent with the model-based predictions, and confirmed that a combinatorial strategy of cobicistat and ibrutinib can be utilized to tailor the systemic exposure to ibrutinib to therapeutically relevant levels (Table 1).

### Pharmacokinetic Model Predictions of Ibrutinib Exposure in Patients with Cancer

**FIGURE 4:**
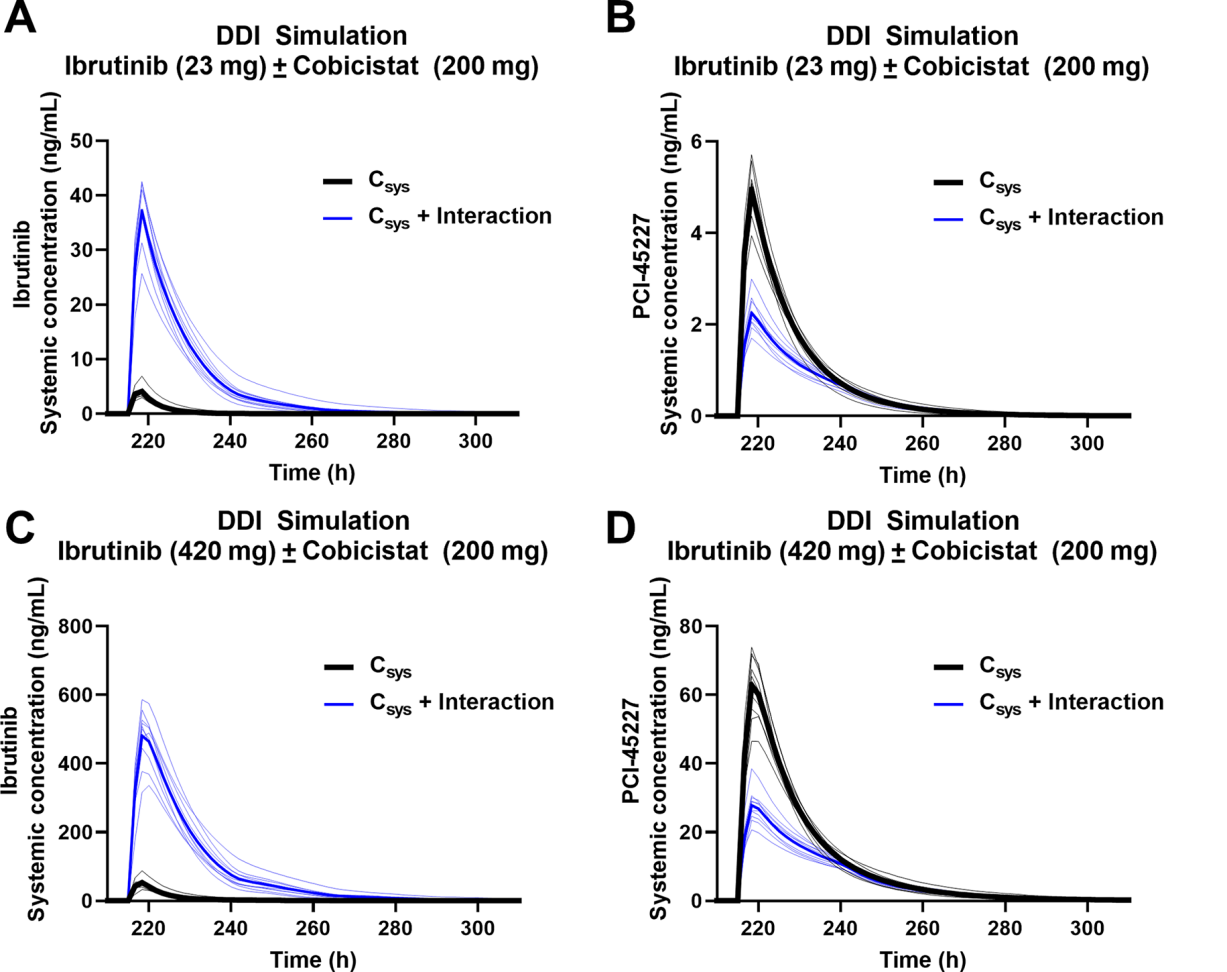
Physiologically based pharmacokinetic modeling to predict effects of cobicistat on ibrutinib levels in humans. Predicted concentrations of ibrutinib (**A**) and PCI-45227 (**B**) after an oral ibrutinib dose of 23 mg administered with or without of cobicistat 200 mg in patients with cancer. Predicted concentrations of ibrutinib (**C**) and PCI-45227 (**D**) after an oral ibrutinib dose of 420 mg administrated with or without cobicistat 200 mg in patients with cancer. Abbreviations: COBI, cobicistat; C_sys_, systemic concentration.

We next applied similar principles to the development of a physiologically based pharmacokinetic model using previously published human data (Fig. 4), which included concentration profiles of ibrutinib and PCI-45227 in fasted healthy individuals following a single oral dose of 420 mg ibrutinib (7), and clinical data associated with cobicistat given at a dose of 200 mg (43). In this model, the absorption phase of ibrutinib was adequately described by assuming that drug remained in solution at all times. The model performance was verified by comparing simulated and observed plasma concentration profiles following multiple oral doses of 420 mg ibrutinib in patients with cancer (44). The final model suggested that cobicistat would cause a 16-fold increase in ibrutinib exposure at regular ibrutinib doses of 420 mg, and predicted that a 23 mg dose of ibrutinib given with a 200 mg dose of cobicistat would achieve a concentration–time profile in patients with cancer similar to that observed following a 420 mg dose of ibrutinib given alone (*P* = 0.99; Table 2). Our model predicted values for the coefficient of variation were 106% for ibrutinib given alone at a dose of 420 mg and 68% for ibrutinib (23 mg) given in combination with cobicistat (*P* = 0.22).

**TABLE 2:** Ibrutinib pharmacokinetic parameters predicted from a physiologically based pharmacokinetic model of drug–drug interaction between ibrutinib and cobicistat.

Ibrutinib dose (mg)	Cobicistat dose (mg)	*T*_max_ (h)	*C*_max_ (ng/mL)	AUC (ng/h/mL)	AUC_inh_/AUC ratio	CL (L/hour)
420	—	1.93 (0.83)	58.4 (53.7)	468 (499)		1,600 (1,167)
420	200	3.02 (1.18)	509 (295)	7,497 (4,934)	20.4 (12.0)	84.5 (60.6)
23	—	1.46 (0.24)	4.77 (4.05)	28 (32)		1,521 (1,129)
23	200	1.80 (0.37)	38.9 (19.5)	476 (326)	22.3 (15.1)	74.6 (54.2)

NOTE: Values are the mean with SD in parenthesis.

Abbreviations: AUC_inh_, AUC in the presence of cobicistat; CL, clearance; *T*_max_, time of maximum plasma concentration.

## Discussion

This study provides support for a growing body of knowledge that inhibitors of CYP3A-mediated metabolism can have a dramatic impact on the pharmacokinetic profile of TKIs, and that this type of drug–drug interaction can be exploited to derive treatment strategies with boosting agents to improve the pharmacologic profile of agents such as ibrutinib. Employing an array of *in vitro* and *in vivo* model systems, ibrutinib was identified as a high-affinity substrate for CYP3A-mediated metabolism, a finding that is consistent with a previous report (13). With the use of CYP3A-deficient mice, we identified cobicistat as a selective and effective agent that can substantially increase the low and erratic oral bioavailability of ibrutinib, while minimizing exposure to the heart, a known site of injury, and without negatively affecting its antileukemic properties. In conjunction with a newly developed, scalable pharmacokinetic model, these findings provide a rationale for the development of a therapeutic strategy in which low-dose ibrutinib can be boosted with cobicistat to reach therapeutically relevant drug levels.

By inhibiting physiologic mechanisms limiting the low oral bioavailability of xenobiotics, pharmacokinetic boosting has facilitated the oral dosing of several anticancer drugs the use of which would otherwise be restricted to intravenous administration (45). This strategy has demonstrated the ability to effectively and safely improve the pharmacokinetic profile of anticancer drugs (14). Recently, there has been a growing interest in the use of this boosting concept as a strategy to improve the pharmacokinetic profile of TKIs already developed and marketed for oral use. Case studies have previously explored the use of cobicistat, to boost levels of TKIs including crizotinib (46) and axitinib (47). Another recent study reported the use of itraconazole, an antifungal drug that is less hepatotoxic than ketoconazole (8), to improve the systemic exposure to ibrutinib in healthy volunteers. In this important proof-of-concept study, the investigators found that itraconazole increased levels of ibrutinib and decreased the interindividual pharmacokinetic variability (from 104% to 55%). This decrease in interindividual pharmacokinetic variability was similar to that seen when ketoconazole was combined with ibrutinib (from 40% to 20%; ref. 7), and consistent with our predicted values. However, the mechanism underlying the reported itraconazole–ibrutinib interaction remains undefined and may involve sites of interaction beyond CYP3A. This would be consistent with the observation that the increase in ibrutinib exposure predicted from a pharmacokinetic model (21-fold; ref. 23) deviates substantially from the experimentally observed 10-fold increase (8), and with prior reports indicating that prototypical inhibitors of CYP3A, such as ketoconazole and itraconazole, can modulate the function of transporters and other drug-metabolizing enzymes of possible relevance to the bioavailability of ibrutinib (29). It should also be pointed out that azole antifungals are associated with significant risk of cardiac dysfunction (33). Given ibrutinib's cardiotoxic potential (48), this suggests that azole antifungals such as itraconazole may not be suitable for chronic administration as CYP3A-boosting drugs in the context of ibrutinib therapy.

The potential ramification of our proposed boosting concept with cobicistat–ibrutinib with respect to potential economic, pharmacokinetic, and pharmacologic benefits requires additional investigation. Nonetheless, it can be envisaged that decreasing the applied ibrutinib dose and associated reduction in costs of treatment could increase drug access. Indeed, ibrutinib is not only more effective than legacy therapies and used by patients for more prolonged periods, but also significantly more expensive per cycle of treatment (49). Despite a growing interest in the development of strategies to decrease the cost of ibrutinib therapy, current approaches remain inadequately characterized and unfit for regular therapy. A recent investigation attempted to decrease ibrutinib cost by sequentially decreasing the amount of ibrutinib capsules taken over time (50). While this strategy appears to successfully achieve changes in pharmacodynamic markers associated with ibrutinib activity, it fails to address the problems surrounding the unpredictable pharmacokinetic profile associated with this drug, which in turn, could potentially result in nonresponders when levels are too low or contribute to idiosyncratic toxicities when certain threshold levels are exceeded. This supposition is consistent with recently reported data from a population pharmacokinetic modeling analysis suggesting that higher plasma concentrations of ibrutinib are associated with a higher rate of discontinuation due to adverse events (42). A properly designed and executed pharmacokinetic boosting strategy can offer significant advantages in this context, and could further decrease the overall cost by facilitating a decrease in the prescribed ibrutinib dose by >18-fold.

In addition to cost considerations, implementation of the boosting concept could also improve the predictability of an individual's pharmacokinetic profile of ibrutinib by decreasing the degree of interindividual variability through a temporal shutting down of the agent's primary pathway of elimination. This expectation is consistent with the well-established inverse relationship between oral bioavailability and interindividual pharmacokinetic variability such that drugs with low bioavailability tend to be associated with greater variability (51). Given that ibrutinib has limited oral bioavailability, strategies to increase ibrutinib's bioavailability should decrease the degree of variability in the drug's pharmacokinetic profile. It should be pointed out that such decreases in variability were not observed in our murine models which were comprised of genetically identical animals of similar health, size, weight, and age, and such observation is consistent with previously reported findings (52).

During the course of our studies, we found that the AUC of ibrutinib achieved with administration of a single oral dose of 30 mg/kg was nearly 6-fold greater than the AUC observed following a dose of 10 mg/kg (Table 1), rather than 3-fold greater as assumed by our population PK model. Regardless, when combined with cobicistat, an ibrutinib dose of 4 mg/kg achieved levels similar to those found with ibrutinib given alone at 30 mg/kg. These unexpected findings suggest that the interaction between ibrutinib and cobicistat is nonlinear and dependent on the ibrutinib dose. Although further studies are required to unravel the mechanistic basis of this dose dependence, the applied murine models provide conceptual support for the proposed strategy to intentionally increase the bioavailability of ibrutinib.

Beyond decreasing pharmacokinetic variability, intentional inhibition of CYP3A activity during therapy with ibrutinib could also independently improve the agent's pharmacodynamic activity. A prior investigation has demonstrated that the combined use of pharmacologic inhibitors of CYP3A with anticancer drugs that are substrates of CYP3A improves the ability of these drugs to kill cancer cells (53). This finding is consistent with our present results demonstrating that cobicistat and ibrutinib act synergistically to kill ibrutinib-sensitive cancer cells. Further research is required to determine the mechanism underlying this synergy, and examine whether this combination is also associated with enhanced antileukemic activity in *in vivo* models. In this context, it is noteworthy that the CYP3A4 enzyme has recently been detected in the bone marrow microenvironment, where it contributes to bone marrow stromal cell–mediated inactivation of and resistance against certain antileukemic TKIs (54). Currently, ongoing studies will focus on examining the potential relevance of this mechanism for efficacy endpoints of the proposed cobicistat–ibrutinib combination *in vivo*.

Overall, our findings signify a critical role for CYP3A-mediated metabolism in the low oral bioavailability of ibrutinib, and demonstrate the possibility to apply boosting with cobicistat to improve the pharmacokinetic profile and therapeutic use of ibrutinib. Future evaluation of this proposed concept in connection with the antileukemic properties of ibrutinib and its application to other TKIs with poor oral bioavailability due to CYP3A-mediated metabolism is warranted.

## Supplementary Material

Supplementary Methods Supplementary methods with additional details regarding methods used for: in vivo pharmacokinetics study drug preparation, in vivo pharmacokinetics study, quantitative measurement of ibrutinib and PCI-45227 concentrations, and microsomal experiments.

Supplementary Figures and Tables Supplementary Figures and Tables including:
Supplementary Figure 1. Plasma concentrations of PCI-45227 in mice.
Supplementary Figure 2. Influence of cobicistat and CYP3A-deficiency on the exposure to ibrutinib after intravenous administration.
Supplementary Figure 3. Metabolism of ibrutinib by liver and intestinal microsomes from wild-type mice.
Supplementary Figure 4. Influence of ketoconazole or cobicistat on the exposure to ibrutinib in male wild-type mice.
Supplementary Figure 5. Transport of ibrutinib by xenobiotic uptake transporters in vitro and in vivo.
Supplementary Figure 6. Influence of cobicistat pre-treatment on the exposure of ibrutinib in heart samples of wild-type mice.
Supplementary Table 1. Pharmacokinetic parameters estimated from a physiologically-based pharmacokinetic model (PBPK).
Supplementary Table 2. Ibrutinib and PCI-45227 pharmacokinetic parameters.
Supplementary Table 3. Population pharmacokinetic model parameters.
