# Supplementary material for: Intentional Modulation of Ibrutinib Pharmacokinetics through CYP3A Inhibition
Source: Cancer Res Commun. 2021 Nov 9;1(2):79–89. doi: 10.1158/2767-9764.CRC-21-0076 (PMC8691714; doi:10.1158/2767-9764.CRC-21-0076)
Supplement: Supplementary Methods — with additional details regarding methods used for: in vivo pharmacokinetics study drug preparation, in vivo pharmacokinetics study, quantitative measurement of ibrutinib and PCI-45227 concentrations, and microsomal experiments. [file crc-21-0076-s01.docx]

**Supplementary Materials**

**Intentional Modulation of Ibrutinib Pharmacokinetics through CYP3A Inhibition**

Eric D. Eisenmann*^1^, Qiang Fu*^1^, Elizabeth M. Muhowski^2^, Yan Jin^1^, Muhammad Erfan Uddin^1^, Dominique A. Garrison^1^, Robert H. Weber^1^, Jennifer Woyach^2^, John C. Byrd^2^, Alex Sparreboom^1^, and Sharyn D. Baker^1^

**Supplementary Methods.**

**Supplemental Methods.**

***In vivo* pharmacokinetics study drug preparation**

Ibrutinib was prepared for oral administration by dissolving powder in DMSO (1%), then adding PEG300 (30%), Tween-80 (1%), and water (68%) stepwise to create a 2 mg/mL suspension. Ibrutinib was prepared for intravenous administration by dissolving powder in DMSO (33%), then adding sterile PBS (66%) to create a 0.2 mg/mL solution. Ketoconazole was prepared for oral administration as a 10-mg/mL suspension in PEG400, and cobicistat as a 6-mg/mL suspension in corn oil.

***In vivo* pharmacokinetics study**

Pharmacokinetic studies were performed as previously described. Briefly, whole blood samples of about 30 μL were collected from each mouse at six time points between 5 min and 8 h after ibrutinib administration. In most cases, samples were collected at 5, 15, and 30 min, and at 1, 3, and 4, 6 or 8 h. The first three samples were obtained from a submandibular vein using a 5 mm Goldenrod sterile animal lancet and heparinized capillary tube. For the fourth and fifth samples, mice were anesthetized under 2% isoflurane and whole blood was taken from the retro-orbital venous plexus using capillary tubes. The final sample was obtained by cardiac puncture using a syringe and needle. Hearts were collected at the terminal time-point and were rinsed with PBS. Blood samples were centrifuged at 13,000 rpm for 5 min and the plasma supernatant was collected. Plasma and organs immediately placed on dry ice, and stored at -80 °C until analysis.

**Quantitative measurement of ibrutinib and PCI-45227 concentrations**

Ibrutinib and PCI-45227 were quantified using a validated UHPLC-MS/MS analytical method. The analytical equipment consisted of a Vanquish UHPLC system, a TSQ Quantum Ultra triple quadrupole mass spectrometer from Thermo Fisher Scientific, and Thermo Trace Finder General Quan system software (version 3.3). An Accucore Vanquish C18 column (100 × 2.1 mm, dp = 1.5 μm; Thermo Fisher Scientific) was protected by a corresponding XBridgeBEH C18, 5-μm guard column. The injection volume of sample was 5.0 μL. The temperature of the autosampler rack was 4 °C, and the temperature of the column was 40 °C. Mobile phase A consisted of water with 0.1% (v/v) formic acid and mobile phase B consisted of acetonitrile: methanol (1:3) with 0.1% (v/v) formic acid. The total run time was 5 min. The mobile phase was delivered at a flow rate of 0.4 mL/min, and the optimized gradient contained time-varying amounts of mobile phase B, as follows: 0-0.5 min, 10% B; 0.5-1.5 min, 95% B; 1.5-2.9 min, 95% B; 2.9-3.0 min, 10% B; and 3.0-5.0 min, 10% B. The mass spectrometric assay settings included a positive voltage applied to the ESI capillary of 3500 V, a capillary temperature of 342 °C, and a vaporizer temperature of 358 °C. Argon was used a collision gas at a pressure of 1.5 mTorr. Precursor molecular ions and product ions for confirmation and detection of ibrutinib (441.24 > 304.0), PCI-45227 (475.3>304.1) and the internal standards [^2^H_5_]-ibrutinib (446.3> 309.0), [^2^H_5_]-PCI-45227 (480.3>309.1). For sample extraction by protein precipitation, 5 µL of plasma, 5 µL of microsomal suspension, or 10 µL of homogenized tissue were added to a tube containing 60 µL of internal standard solution and 20 µL of methanol. Samples were then vortex-mixed, centrifuged at 13,000 rpm for 9.5 min, and supernatants collected and stored frozen until analysis. Results from assay validation studies revealed that the within-day precision and between-day precision ranged 4.52-10.7%, and values for accuracy were 95.2-109%, respectively. The lower limit of quantification was 5 ng/mL, using 5-µL sample volumes.

**Microsomal experiments**

Standard procedures were used to isolate microsomes from liver and duodenum. In brief, ~100 mg tissue was homogenized using a buffer containing 100 mM Tris-Base, 100 mM KCl, 1 mM EDTA, and 20 µM butylated hydroxytoluene, and then centrifuged using a JA-20.1 rotor at 12,000 rpm for 15 min. The supernatant was transferred to a 70.Ti rotor and ultracentrifuged at 34,000 rpm for 1 h, after which the pellet was collected and re-suspended in a microsomal storage buffer containing 100 mM potassium phosphate, 1 mM EDTA, 20% glycerol, 1 mM dithiothreitol, and 20 µM butylated hydroxytoluene. Protein concentrations were determined using a Piece BCA Protein Assay Kit.
