# Supplementary material for: Intentional Modulation of Ibrutinib Pharmacokinetics through CYP3A Inhibition
Source: Cancer Res Commun. 2021 Nov 9;1(2):79–89. doi: 10.1158/2767-9764.CRC-21-0076 (PMC8691714; doi:10.1158/2767-9764.CRC-21-0076)
Supplement: Supplementary Figures and Tables — including: Supplementary Figure 1. Plasma concentrations of PCI-45227 in mice. Supplementary Figure 2. Influence of cobicistat and CYP3A-deficiency on the exposure to ibrutinib after intravenous administration. Supplementary Figure 3. Metabolism of ibrutinib by liver and intestinal microsomes from wild-type mice. Supplementary Figure 4. Influence of ketoconazole or cobicistat on the exposure to ibrutinib in male wild-type mice. Supplementary Figure 5. Transport of ibrutinib by xenobiotic uptake transporters in vitro and in vivo. Supplementary Figure 6. Influence of cobicistat pre-treatment on the exposure of ibrutinib in heart samples of wild-type mice. Supplementary Table 1. Pharmacokinetic parameters estimated from a physiologically-based pharmacokinetic model (PBPK). Supplementary Table 2. Ibrutinib and PCI-45227 pharmacokinetic parameters. Supplementary Table 3. Population pharmacokinetic model parameters. [file crc-21-0076-s02.docx]

**Intentional Modulation of Ibrutinib Pharmacokinetics through CYP3A Inhibition**

Eric D. Eisenmann*^1^, Qiang Fu*^1^, Elizabeth M. Muhowski^2^, Yan Jin^1^, Muhammad Erfan Uddin^1^, Dominique A. Garrison^1^, Robert H. Weber^1^, Jennifer Woyach^2^, John C. Byrd^2^, Alex Sparreboom^1^, and Sharyn D. Baker^1^

**Supplementary Figure 1.** **Plasma concentrations of PCI-45227 in mice.**

**Supplementary Figure 2. Influence of cobicistat and CYP3A-deficiency on the exposure to ibrutinib after intravenous administration.**

**Supplementary Figure 3.** **Metabolism of ibrutinib by liver and intestinal microsomes from wild-type mice.**

**Supplementary Figure 4. Influence of ketoconazole or cobicistat on the exposure to ibrutinib in male wild-type mice.**

**Supplementary Figure 5. Transport of ibrutinib by xenobiotic uptake transporters *in vitro*** **and *in vivo*.**

**Supplementary Figure 6. Influence of cobicistat pre-treatment on the exposure of ibrutinib in heart samples of wild-type mice.**

**Supplementary Table 1. Pharmacokinetic parameters estimated from a physiologically-based pharmacokinetic model (PBPK).**

**Supplementary Table 2. Ibrutinib and PCI-45227 pharmacokinetic parameters.**

**Supplementary Table 3. Population pharmacokinetic model parameters.**

**Supplementary Figure 1. Plasma concentrations of PCI-45227 in mice.** Plasma concentrations of PCI-45227 in wild-type mice receiving oral ibrutinib (10 mg/kg) 30 min after oral ketoconazole (50 mg/kg) (**A**) or cobicistat (30 mg/kg) followed by a single dose of ibrutinib (**B**) or after 5 consecutive daily doses (**C**). Data presented represent the mean (symbols) ± SEM (error bars) of 4-10 observations per group.

**Supplementary Figure 2. Influence of cobicistat and CYP3A-deficiency on the exposure to ibrutinib after intravenous administration.** Plasma concentration-time curves of **(A)** ibrutinib and **(B)** PCI-45227 in female wild-type and CYP3A(-/-) FVB mice administered ibrutinib 1 mg/kg intravenously, 30 min after oral administration of cobicistat 30 mg/kg or corn oil vehicle (n=5/group). Concentrations of ibrutinib and PCI-45227 were determined by LC-MS/MS.

**Supplementary Figure 3.** **Metabolism of ibrutinib by liver and intestinal microsomes from wild-type mice.** Microsomes were isolated from **(A)** liver and **(B)** intestine from wild-type and CYP3A(-/-) mice and incubated with 10 μM ibrutinib for 1 h. Concentrations of ibrutinib and PCI-45227 in tissue microsomes were determined by LC-MS/MS.

**Supplementary Figure 4. Influence of ketoconazole or cobicistat on the exposure to ibrutinib in male wild-type mice.** Plasma concentration-time curves of ibrutinib and PCI-45227 in wild-type male FVB mice administered ibrutinib 10 mg/kg orally (PO) **(A)** 30 min after ketoconazole 50 mg/kg PO (n=4), PEG400 vehicle PO (n=5), or no vehicle (n=5); or **(B)** cobicistat 30 mg/kg PO (n=5), corn oil vehicle PO or no vehicle (n=5). Concentrations of ibrutinib and PCI-45227 were determined in plasma by LC-MS/MS.

**Supplementary Figure 5. Transport of ibrutinib by xenobiotic uptake transporters *in vitro*** **and *in vivo*.** Uptake of radiolabeled **(A)** prototypical substrates or **(B)** ibrutinib in cells overexpressing transporters. Values are presented as fold change compared to cells expressing empty vector constructs (n=6 across 2 biological replicates). Ibrutinib concentration-time profiles in **(C)** female or **(D)** male C57BL/6 wild-type or OATP2B1(-/-) mice; or **(E)** female DBA wild-type or OATP1B2(-/-) mice treated with ibrutinib 10 mg/kg orally (n=5/group). Concentrations of ibrutinib and PCI-45227 were quantitated in plasma by LC-MS/MS.

**Supplementary Figure 6. Influence of cobicistat pre-treatment on the exposure of ibrutinib in heart samples of wild-type mice.** Heart concentrations of ibrutinib in female wild-type mice at six hours after administration of ibrutinib 10mg/kg orally, 30 min after oral administration of cobicistat 30mg/kg (n=5) or corn oil vehicle (n=5). Heart samples were homogenized and ibrutinib concentrations were determined by LC-MS/MS and normalized to protein and plasma concentrations. An unpaired t-test was used to compare the mean heart-to-plasma ratio between vehicle- and cobicistat-treated mice. *** P<0.001

**Supplementary Table 1. Pharmacokinetic parameters estimated from a physiologically-based pharmacokinetic model (PBPK).**

| Parameter and models | Ibrutinib | PCI-45227 | Cobicistat |
| --- | --- | --- | --- |
| Molecular weight | 441 | 475 | 776 |
| Log P_o:w_ | 3.97 | 2.21 | 4.36 |
| pKa | 3.78 | 4.06 | 6.69 |
| B/P ratio | 0.827 | 0.790 | 0.55 |
| fu, plasma | 0.027 | 0.056 | 0.020 |
| Caco-2 (10^-6 cm/s) | 32.6 | -- | Ka (1/h)= 0.29 |
| Distribution model | Minimal PBPK | Minimal PBPK | Minimal PBPK |
| V_ss_ (L/kg) | 3.80 | 9.0 | 0.46 |
| Clearance type | Enzyme Kinetics | In Vivo Clearance | In Vivo Clearance |
| CYP3A4 Clint (µL/min/mg) | 8312 | Clpo (L/h) : 433 | Clpo (L/h) : 14 |
| HLM Other Clint (µL/min/mg) | 364 |  |  |
| Clr (L/h) | 0.0037 |  |  |

Note: Model parameters were taken from [17, 18] and Drugbank.

*Abbreviations****:*** Log P_o:w_, octanol/water partition coefficient; pKa, acid dissociation constant; B/P ratio, blood to plasma ratio; fu plasma, unbound drug in the plasma; Vss, steady-state volume of distribution; HLM, human liver microsomes; Clr, renal clearance; Clint, intrinsic clearance; HLM, human liver microsomes; CLpo, oral clearance.

**Supplementary Table 2. Ibrutinib and PCI-45227 pharmacokinetic parameters.**

| Mouse Genotype | Ibrutinib Dose (mg/kg) | Ibrutinib Route of Administration | Co-treatment | Sex | Ibrutinib Cmax (ng/mL) | Ibrutinib AUC(0-last) (ng*hr/mL) | Ibrutinib AUC Fold Increase | PCI-45227 Cmax (ng/mL) | PCI-45227 AUC(0-last) (ng*hr/mL) | PCI-45227: Parent AUC Ratio |
| --- | --- | --- | --- | --- | --- | --- | --- | --- | --- | --- |
| Wild-type FVB | 1 | IV | Corn Oil | F | 1414 (52) | 521 (17) | - | 197 (5) | 311 (15) | 0.40 |
| Wild-type FVB | 1 | IV | Cobicistat | F | 1545 (58) | 1007 (92)** | 1.9 | 25 (11) | 38 (27) | 0.04 |
| CYP3A(-/-) | 1 | IV | Corn Oil | F | 1860 (147) | 1172 (76)** | - | ND | ND | ND |
| CYP3A(-/-) | 1 | IV | Cobicistat | F | 1438 (132) | 895 (49)** | 0.8 | ND | ND | ND |
| Wild-type FVB | 10 | PO | None | M | 404 (91) | 429 (62) | - | 333 (112) | 1080 (153) | 2.5 |
| Wild-type FVB | 10 | PO | PEG400 | M | 348 (82) | 636 (102)^a^ | 1.5 | 293 (69) | 929 (164) | 1.5 |
| Wild-type FVB | 10 | PO | Ketoconazole | M | 1256 (258)** | 6643 (1383)*** | 9.9 | 236 (37) | 911 (94) | 0.14 |
| Wild-type FVB | 10 | PO | No Vehicle | M | 360 (49) | 520 (43) | - | 578 (100) | 1463 (83) | 2.8 |
| Wild-type FVB | 10 | PO | Corn Oil | M | 243 (67) | 663 (185)^a^ | 1.3 | 308 (70) | 1124 (152)^a^ | 1.7 |
| Wild-type FVB | 10 | PO | Cobicistat | M | 1356 (491)* | 4335 (393)*** | 6.5 | 124 (34)* | 417 (60)*** | 0.096 |
| Wild-type C57BL/6 | 10 | PO | None | F | 1420 (144) | 1454 (202) | - | 1373 (70) | 3007 (236) | 2.1 |
| OATP2B1(-/-) | 10 | PO | None | F | 1247 (179) | 1746 (328) | 1.2 | 745 (115)** | 1952 (168)** | 1.1 |
| Wild-type C57BL/6 | 10 | PO | None | M | 1382 (407) | 1388 (77) | - | 1252 (97) | 3338 (239) | 2.4 |
| OATP2B1(-/-) | 10 | PO | None | M | 1631 (212) | 1773 (359) | 1.3 | 850 (96)* | 2236 (160)*** | 1.3 |
| Wild-type DBA | 10 | PO | None | F | 1964 (559) | 2725 (662) | - | 234 (47) | 343 (52) | 0.12 |
| OATP1B2(-/-) | 10 | PO | None | F | 2169 (654) | 2100 (314) | 0.8 | 157 (9) | 304 (12) | 0.14 |

Values are the mean with standard deviation in parenthesis.

Treatment groups involved 4-5 mice per experiment.

*Abbreviations:* F, female; M, male; Cmax, maximum plasma concentration; AUC(0-last), area under the concentration-time curve (AUC) from time zero to the last observed timepoint; ND, not determined (concentrations were below the analytical assay lower limit of quantitation). *P<0.05, **P<0.01, ***P<0.001, ^a^not significant

**Supplementary Table 3. Population pharmacokinetic model parameters.**

| Parameter | Estimate | % RES | BE (95% CI) | |
| --- | --- | --- | --- | --- |
| CL/F (mL/h) | 1.87 | 45.4 | 1.17 | 3.10 |
| V_c_ (mL) | 0.102 | 0.132 | 0.102 | 0.107 |
| K_a_ (h^-1^) | 0.340 | 5.34 | 0.254 | 0.428 |
| Q/F (mL/h) | 0.0293 | 1.21 | 0.010 | 0.0477 |
| V_m_ (mL) | 0.0578 | 4.15 | 0.0147 | 0.199 |
| Lag (hr) | 0.0324 | 1.43 | 0.000786 | 0.0568 |
| V_p_ (mL) | 0.116 | 2.75 | 0.103 | 0.197 |
| CL_m_ (mL/h) | 1.98 | 4.0 | 1.83 | 1.99 |
| F_met_ | 0.0216 | 0.569 | 0.0103 | 0.0348 |
| CYP3A | 6.80 | 165 | 3.25 | 9.93 |
| IIV |  |  |  |  |
| CL/F | 0.108 | 7.42 | 0.0203 | 0.277 |
| V_c_ | 0.167 | 21.3 | 1.29E-05 | 0.549 |
| K_a_ | 0.209 | 19.3 | 2.88E-05 | 0.624 |
| Q/F | 0.0009 | 0.0233 | 6.61E-05 | 0.00103 |
| V_m_ | 1.38 | 40.0 | 0.837 | 2.45 |
| Lag | 0.0264 | 4.46 | 1.00E-06 | 0.117 |
| V_p_ | 0.295 | 54.7 | 7.13E-05 | 1.51 |
| CL_m_ | 0.0242 | 9.44 | 1.50E-07 | 0.378 |
| RV |  |  |  |  |
| IBU | 0.425 | 6.68 | 0.319 | 0.551 |
| PCI | 0.730 | 28.2 | 0.238 | 0.110 |

*Abbreviations:* %CV, coefficient of variation; BE, backward elimination; CI, confidence interval; CL/F, total clearance; CLm, PCI-45227 clearance; Fmet, metabolism of PCI-45227; IIV, inter-individual variability; Ka, absorption rate constant; lag, lag time; Q/F, inter compartmental clearance; RV, residual variability; RSE, residual standard error; Vc, volume of distribution for the central compartment of ibrutinib; Vp, volume of distribution for the peripheral compartment of ibrutinib; Vm, volume of distribution for the central compartment of PCI-45227; IBU, Ibrutinib; PCI, PCI-452270
